# Quantifying income inequality in years of life lost to COVID-19: a prediction model approach using Dutch administrative data

**DOI:** 10.1093/ije/dyad159

**Published:** 2023-12-11

**Authors:** Jawa Issa, Bram Wouterse, Elena Milkovska, Pieter van Baal

**Affiliations:** Erasmus School of Health Policy and Management, Erasmus University Rotterdam, Rotterdam, The Netherlands; Erasmus School of Health Policy and Management, Erasmus University Rotterdam, Rotterdam, The Netherlands; Erasmus School of Health Policy and Management, Erasmus University Rotterdam, Rotterdam, The Netherlands; Erasmus School of Health Policy and Management, Erasmus University Rotterdam, Rotterdam, The Netherlands

**Keywords:** COVID-19, income inequality, The Netherlands, years of life lost, mortality

## Abstract

**Background:**

Low socioeconomic status and underlying health increase the risk of fatal outcomes from COVID-19, resulting in more years of life lost (YLL) among the poor. However, using standard life expectancy overestimates YLL to COVID-19. We aimed to quantify YLL associated with COVID-19 deaths by sex and income quartile, while accounting for the impact of individual-level pre-existing health on remaining life expectancy for all Dutch adults aged 50+.

**Methods:**

Extensive administrative data were used to model probability of dying within the year for the entire 50+ population in 2019, considering age, sex, disposable income and health care use (*n *= 6 885 958). The model is used to predict mortality probabilities for those who died of COVID-19 (had they not died) in 2020. Combining these probabilities in life tables, we estimated YLL by sex and income quartile. The estimates are compared with YLL based on standard life expectancy and income-stratified life expectancy.

**Results:**

Using standard life expectancy results in 167 315 YLL (8.4 YLL per death) which is comparable to estimates using income-stratified life tables (167 916 YLL with 8.2 YLL per death). Considering pre-existing health and income, YLL decreased to 100 743, with 40% of years lost in the poorest income quartile (5.0 YLL per death). Despite individuals in the poorest quartile dying at younger ages, there were minimal differences in average YLL per COVID-19 death compared with the richest quartile.

**Conclusions:**

Accounting for prior health significantly affects estimates of YLL due to COVID-19. However, inequality in YLL at the population level is primarily driven by higher COVID-19 deaths among the poor. To reduce income inequality in the health burden of future pandemics, policies should focus on limiting structural differences in underlying health and exposure of lower income groups.

Key Messages

- Individuals with a lower income and underlying health problems were more likely to suffer the fatal consequences of COVID-19.
- Using standard remaining life expectancy leads to an overestimation of years of life lost, which previous studies accounting for health status have estimated to range between 1.3 and 3.9 years per COVID-19 death depending on the indicators of health status included.
- Combining individual administrative data for the entire Dutch population on cause of death, income and health care use to produce estimates of years of life lost that take prior health status into account, reflects substantial inequality in the disease burden of COVID-19 deaths in The Netherlands in 2020 by income, with the lowest income group bearing 40% of the total of 100 743 years of life lost.
- To reduce income inequality in the health burden of future pandemics, policies should focus on limiting structural differences in underlying health and limiting the exposure of lower income groups.

## Introduction

There is consistent evidence that individuals with a low socioeconomic status (SES) have been more severely affected by the COVID-19 pandemic,^1^ and an unequal distribution of COVID-19 mortality has been found in many countries.^2^^,^^3^ This unequal distribution of COVID-19 mortality also suggests that the disease burden from COVID-19 mortality is highest for the poorest SES groups.^4–6^ However, this is not necessarily the case as it also depends on the remaining life expectancy of those who die of COVID-19.

Estimates of the health burden of COVID-19 tend to rely on years of life lost (YLL): the expected number of years that individuals who die of COVID-19 would otherwise have lived. YLL are traditionally based on standard remaining life expectancy by age and sex,^7–9^ ignoring differences in longevity among various health and income groups.^10^ Considering that individuals who die of COVID-19 often have underlying health problems such as cancer, chronic kidney disease, diabetes mellitus and hypertension,^11–13^ using population life expectancy leads to an overestimation in YLL. Previous studies suggest that this overestimation ranges between 1.3 and 3.9 years of life per COVID-19, death depending on the indicators of health status used to account for underlying health problems.^14–17^ Some of these studies have made use of individual-level data and considered pre-existing health conditions but only take into account a limited amount of health indicators,^14^ or were not able to use comparable datasets for predicting survival and estimating pre-existing health status for those who died of COVID-19.^15^^,^^16^

Adjusting for pre-existing health problems likely matters for estimates of socioeconomic inequality in YLL due to COVID-19. People with a low income have on average a lower life expectancy than people with a higher income.^18^ The income gradient in COVID-19 deaths is partly driven by the relatively large number of individuals in poor health among lower income groups being susceptible to dying from COVID-19.^19–21^ Studies on socioeconomic differences in YLL due to COVID-19, based on standard estimates of life expectancy (not taking prior health into account), find higher YLL among the poor. These results are, by construction, entirely driven by the differences in the number of COVID-19 deaths and the age composition of deaths across SES groups.^4–6^ Thus far, no study has shown the income differences in YLL while taking both pre-existing health and income into account. The aim of this paper is to quantify the YLL associated with COVID-19 deaths in 2020 in The Netherlands for each sex and income group, while accounting for pre-existing differences in health. We do this using microdata on cause of death, income and health care use for the entire Dutch 50+ population.

## Methods

### Study design

We estimated the number of life-years lost per COVID-19 death in two steps. The first step is the estimation of a prediction model for the annual mortality probability in 2019 as a function of age, sex, disposable income and health care use, based on the entire Dutch 50+ population alive on 1 January 2019 (more than 7 million observations). The second step is the identification of all Dutch individuals aged 50+ who died of COVID-19 in 2020. We then used the distribution of covariates among these individuals and the prediction model to predict counterfactual mortality probabilities, and combined these in period life tables to calculate life-years lost; see Supplementary Data (available as Supplementary data at *IJE* online) for details on the sample selection.

### Source data

We obtained administrative records covering information on all Dutch residents from 2018 to 2020. Centraal Bureau voor de Statistiek (Statistics Netherlands) provided access to individual-level data on cause of death, age, sex, disposable income and health care use. Using pseudonymized personal identification numbers for all residents, the data sources were linked at the individual level. Data from the Dutch municipality registry provides information on age and sex. Age on 1 January of the studied year was categorized into single-year ages and a 100+ category. As a measure of income, we used annual disposable household income (corrected for the size and composition of the household^22^) obtained from the tax registry. As a measure of pre-existing health status, we used data on outpatient medication use and nursing home admissions.

The data on medication use contain information on the types of outpatient medication used per year which were reimbursed by the Dutch compulsory social health insurance. Medications are classified using the fourth edition of the World Health Organization (WHO) Anatomical Therapeutic Chemical classification codes (ATC-4). Considering that we do not need to distinguish between drugs’ pharmacological and chemical properties, we chose to keep the model parsimonious by accounting for the drugs’ therapeutic subgroup instead. As such, we used two-digit ATC-4 codes, resulting in a total of 86 variables. For nursing home admissions, information is made available by the Centraal Administratie Kantoor (Central Administrative Office) and contains the start and end date of each stay in a nursing home. Since most clients remain in a nursing home for the rest of their lives, we created a variable indicating whether an individual had used any nursing home care during the prior year.

The Dutch cause of death registry contains the date and underlying main cause of death classified by the 10th revision of the WHO International Statistical Classification of Diseases (ICD-10 codes). Codes U07.1 (confirmed COVID-19) and U07.2 (suspected COVID-19, 13.2% of total) are used to identify COVID-19 deaths.^23^ In The Netherlands, the medical practitioner who attended to the deceased must complete the cause of death certificate. Usually it is the attending physician, but sometimes it can be another physician or a coroner. The completeness of records is checked with the Personal Records Database, and correspondence is held with the responsible certifier regarding unclear or incomplete cause of death certificates.^24^

### Statistical analysis

#### Prediction of individual-level probability of death

To estimate the prediction model for the counterfactual annual mortality probability, we used data preceding the COVID-19 pandemic. We estimated the probability of dying in 2019 for the entire 50+ population alive at the start of that year, using a logit model. We included the following predictors: age (single-year dummies), logged income and medication use (ATC-4 second-level dummies). Age was measured at the start of the year, and for income and medication we used lagged (2018) values. We ran four separate models based on sex and whether individuals lived a nursing home in 2018.

Using the estimated coefficients, predictions of the probability of dying in 2020 were made for each individual who died of COVID-19 in 2020. For the predictions, we used age at the start of 2020 and the lagged (2019) values of income, medication use and nursing home admissions.

#### Calculation of life table by sex and income quartile

To estimate remaining life expectancies, we averaged the predicted annual age-specific mortalities among the individuals who died from COVID-19 by sex and income quartile, and used cubic splines to smooth the resulting age patterns. As income can vary throughout life and by sex, we opted for a relative measure of the income position, using age- and sex-specific income quartiles (Supplementary Figure S2, available as Supplementary data at *IJE* online). This also allows for a convenient comparison of the outcomes across sex and income groups, as their age composition is the same. We then calculated sex- and income quartile-specific period life tables (thus eight in total) using the average age-specific predicted mortality probabilities by sex and income quartile as inputs (see Supplementary Methods, available as Supplementary data at*IJE* online, for details).

#### Calculation of YLL by sex and income quartile

Total YLL for each age-sex-income subgroup were calculated by multiplying the number of COVID-19 deaths within each group by the age-sex-income-specific life expectancy. Consequently, we aggregated by sex and income quartile by summing YLL across ages. We also calculated average YLL per COVID-19 death per sex and income group by dividing the total YLL by the total number of COVID-19 deaths per group. A similar process was used to compare results with income-stratified remaining life expectancy (stratified by age, sex and income) and standard remaining life expectancy (stratified by age and sex only—see Supplementary Methods, available as Supplementary data at *IJE* online, for details).

## Results

First, we show the distribution of average age at death, medication usage and nursing home usage across sex and income groups (Table 1). Some differences between the various sex and income groups include a higher average age at death for richer income quartiles, lower number of medication users and a lower nursing home usage. Moreover, women have on average a higher age at death and a higher nursing home usage.

**Table 1.** Sample description for the 2019 and 2020 Dutch resident population aged 50+ years, by sex and income quartile

	1st quartile (poorest)	2nd quartile	3rd quartile	4th quartile (richest)	Total
**2019 population sample aged 50+ years**					
Men					
Average age at death (all-cause mortality), years	75.4	77.7	78.6	79.1	77.3
Medication usage^a^	651 406 (19.7)	662 502 (20.0)	653 879 (19.7)	639 350 (19.3)	2 607 137 (78.6)
Nursing home usage^a^	35 855 (1.1)	9022 (0.3)	7397 (0.2)	5243 (0.2)	57 517 (1.7)
Total^a^	828 837 (25.0)	828 725 (25.0)	828 718 (25.0)	828 700 (25.0)	3 314 980 (100)
Women					
Average age at death (all-cause mortality), years	80	81	82	83	81.3
Medication usage	730 872 (20.5)	753 855 (21.1)	745 385 (20.9)	729 688 (20.4)	2 959 800 (82.9)
Nursing home usage	57 215 (1.6)	18 209 (0.5)	15 574 (0.4)	13 415 (0.4)	104 413 (2.9)
Total	892 834 (25.0)	892 760 (25.0)	892 702 (25.0)	892 682 (25.0)	3 570 978 (100)
**2020 population sample aged 50+ years**					
Men					
Average age at death (all-cause mortality), years	75.8	77.9	78.7	79.4	77.6
Medication usage	662 122 (19.6)	672 741 (19.9)	663 703 (19.6)	649 022 (19.2)	2 647 588 (78.4)
Nursing home usage	36 612 (1.1)	9232 (0.3)	7411 (0.2)	5397 (0.2)	58 652 (1.7)
Total	844 555 (25.0)	844 472 (25.0)	844 438 (25.0)	844 416 (25.0)	3 377 881 (100)
Women					
Average age at death (all-cause mortality), years	80.3	81.3	82.4	83.2	81.5
Medication usage	741 244 (20.4)	762 340 (21.0)	755 072 (20.8)	738 511 (20.3)	2 997 167 (82.6)
Nursing home usage	57 368 (1.6)	18 641 (0.5)	15 637 (0.4)	13 149 (0.4)	104 795 (2.9)
Total	907 489 (25.0)	907 369 (25.0)	907 378 (25.0)	907 320 (25.0)	3 629 556 (100)

a Number in brackets is the percentage within sex-group.

**Figure 1.**
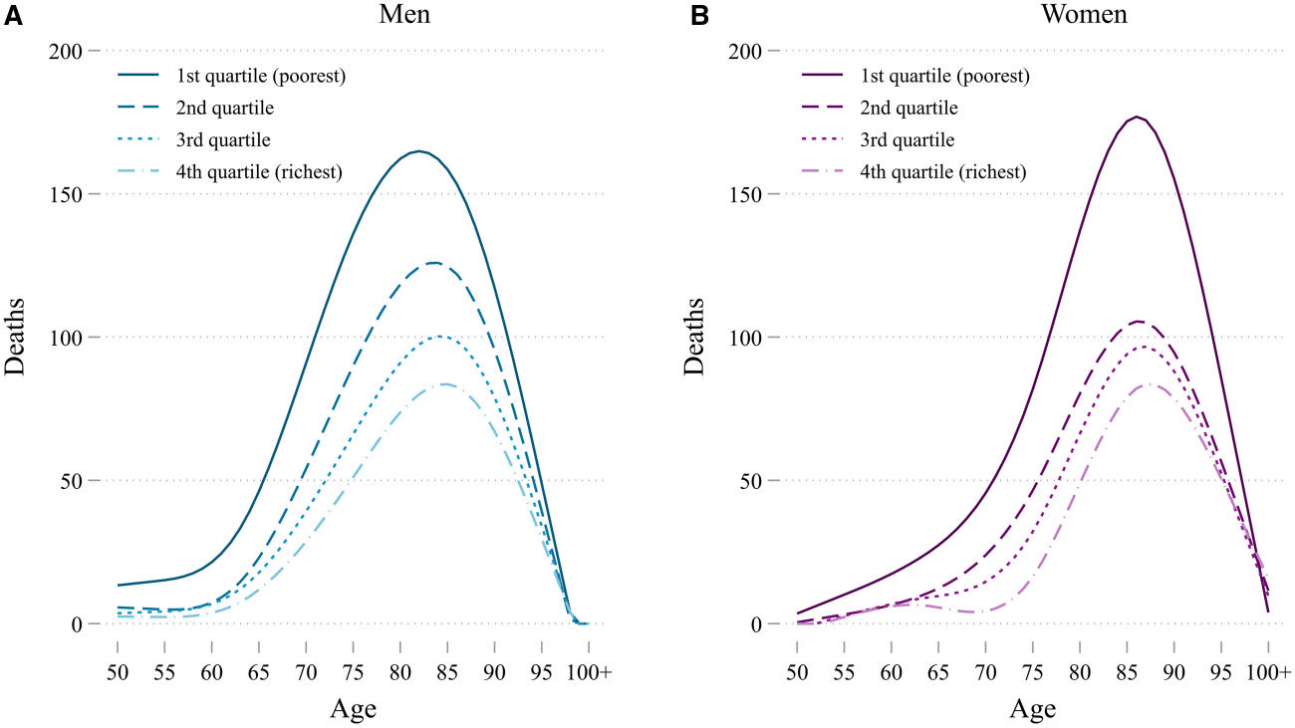
COVID-19 deaths in The Netherlands in 2020 by age for men (A) and women (B). Numbers are smoothed using cubic splines in accordance with anonymity requirements of Statistics Netherlands (actual numbers are used in the analysis)

In Figure 1, we present the distribution of COVID-19 deaths in 2020 in The Netherlands by age for each sex and income group. The peak of COVID-19 deaths occurs just below 85 years of age for men and just over 85 years of age for women. There is a clear income gradient in COVID-19 deaths, with the number of deaths decreasing with higher income at every age.

**Figure 2.**
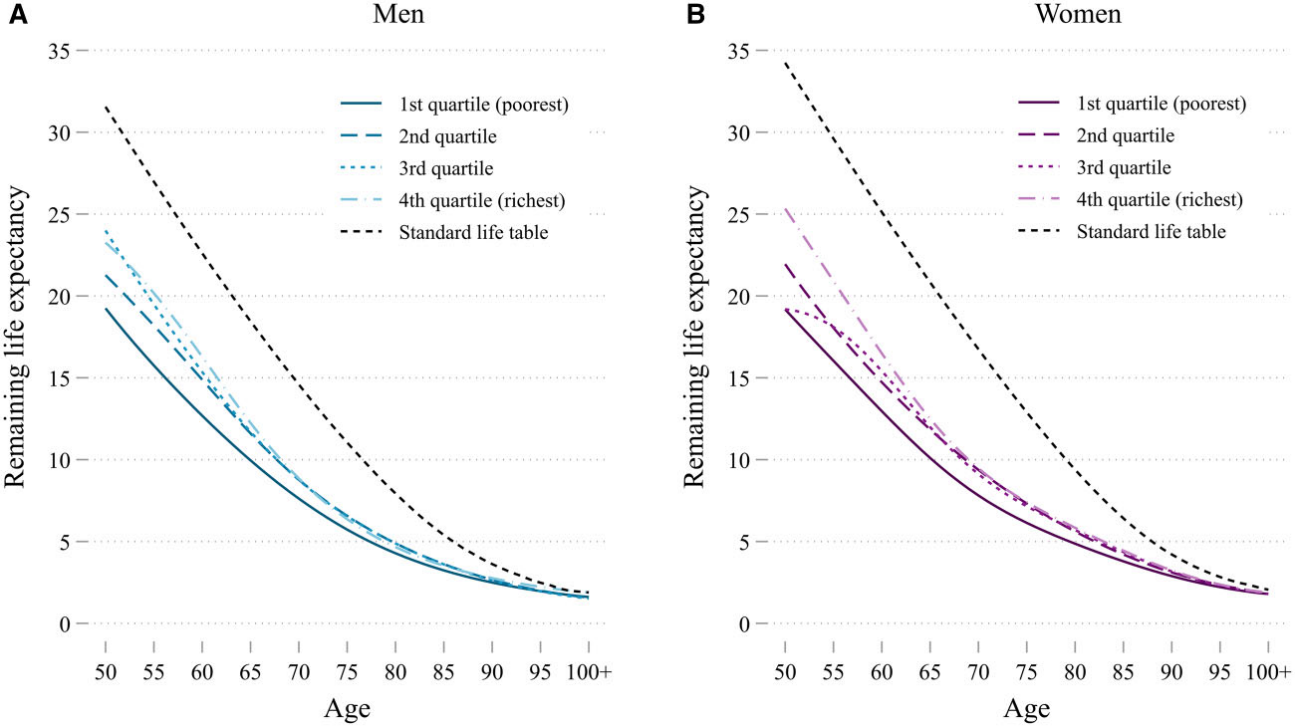
Remaining life expectancy as calculated using our prediction approach for individuals who died of COVID-19 in The Netherlands in 2020, presented alongside standard sex-specific estimates for residents of The Netherlands in 2019. Numbers are smoothed using cubic splines in accordance with anonymity requirements of Statistics Netherlands (actual numbers are used in the analysis)

The second input for our YLL estimates is the remaining life expectancy (Figure 2) which is derived from mortality probabilities (see Supplementary Figure S3, available as Supplementary data at *IJE* online) of individuals who died from COVID-19, as predicted by the regression model averaged by sex and income quartile (details on the regression models can be found in Supplementary Table S1, available as Supplementary data at *IJE* online). With the exception of the poorest income quartile, for which the predicted mortality probabilities are highest, there is relatively little difference across income quartiles. The estimated remaining life expectancies of those who died from COVID-19 by age, sex and income are shown in Figure 2 and, as a reference, the figure also displays the remaining life expectancies based on age and sex only—similar to standard life expectancy as reported by Statistics Netherlands.^25^ For all sex-income groups, the estimated remaining life expectancies of those who died of COVID-19 are below the standard life expectancy, indicating that individuals who died from COVID-19 were, on average, in poorer health than the rest of the population. The estimated life expectancy for individuals in the poorest income quartile is clearly lower at every age (and for both men and women) than for individuals in the other income quartiles; see Supplementary Figure S4 (available as Supplementary data at *IJE* online) for the added intermediate income-stratified analysis.

**Figure 3.**
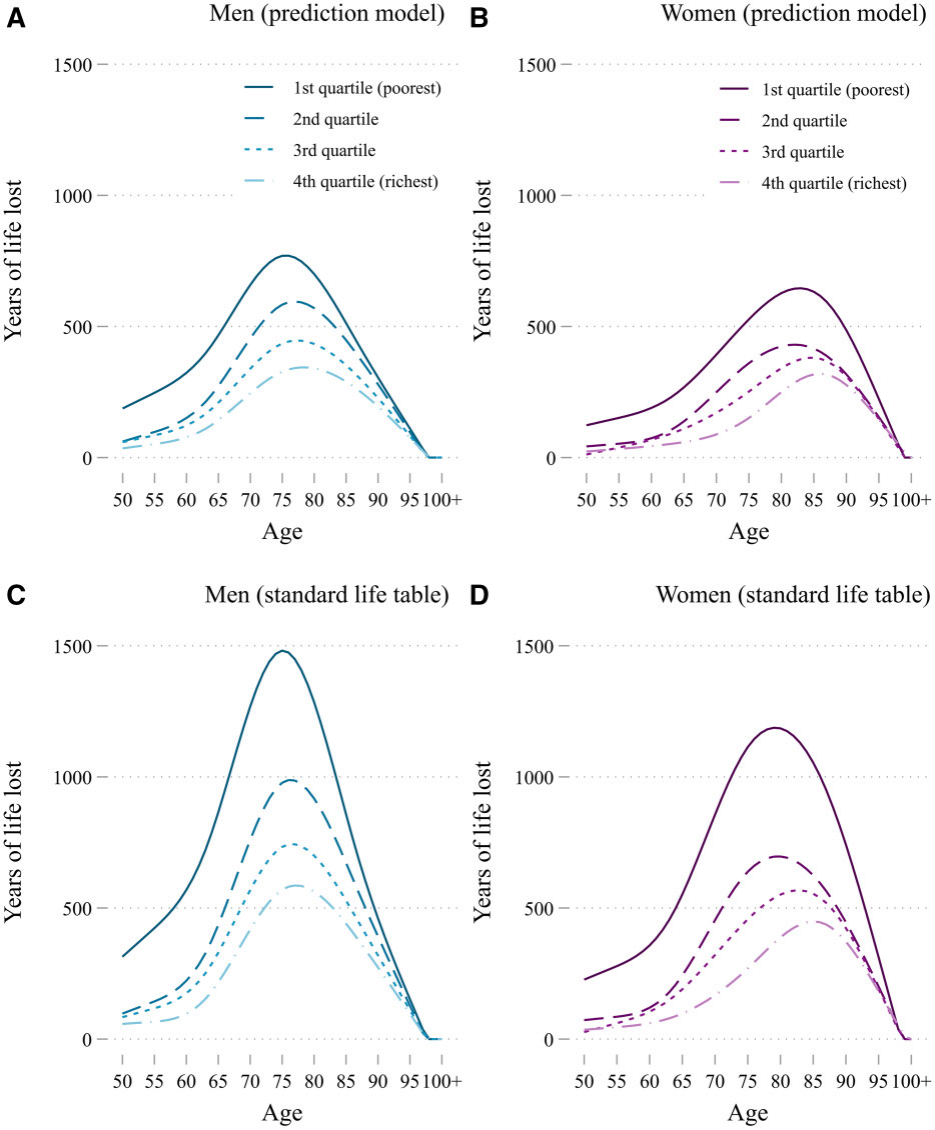
Years of life lost to COVID-19 for the 50+ population as calculated using our prediction approach (A, B) and using standard sex-specific remaining life expectancy (C, D) in The Netherlands in 2020. Numbers are smoothed using cubic splines in accordance with anonymity requirements of Statistics Netherlands (actual numbers are used in the analysis)

In Figure 3, we show the total YLL due to COVID-19 deaths by age for each sex-income group. Panels A and B show a strong income gradient, with YLL decreasing with income for each age and sex. In panels C and D, we used standard remaining life expectancy to calculate YLL, which resulted in higher estimates of YLL but similar income and age patterns; see Supplementary Figure S5 (available as Supplementary data at *IJE* online) for the added intermediate income-stratified analysis.

A summary of all results for each sex and income group is shown in Table 2. Using the prediction model, the average YLL per COVID-19 death is 5.3 for men and 4.8 for women. The average age at death for men/women who die of COVID-19 shows a 2.6/3.4-year difference between the poorest and richest quartile. Despite this difference, the average YLL are similar across income groups. This implies that the lower average age at death for the poorest income groups is offset by the lower life expectancy by age (see Figure 2).

**Table 2.** COVID-19 deaths, average age at death and total and average years of life lost to COVID-19 for the population aged 50+ years, by sex and income quartile in The Netherlands in 2020

			**Years of life lost** ^a^			Average years of life lost		
Sex and income quartile	**Number of COVID-19 deaths** ^a^	Average age at death (years)	Life tables based on prediction model	Income-stratified life tables	Standard life tables	Life tables based on prediction model	Income-stratified life tables	Standard life tables
Men								
1st quartile (poorest)	3976 (37.3)	79	20 886 (37.4)	33 663 (37.3)	37 502 (40.6)	5.3	8.5	9.4
2nd quartile	2757 (25.9)	80.5	14 769 (26.4)	23 111 (25.6)	23 251 (25.2)	5.4	8.4	8.4
3rd quartile	2173 (20.4)	80.9	11 479 (20.5)	18 503 (20.5)	17 959 (19.4)	5.3	8.5	8.3
4th quartile (richest)	1744 (16.4)	81.6	8741 (15.6)	15 001(16.6)	13 697 (14.8)	5	8.6	7.9
Total	10 650 (100)	80.2	55 875 (100)	90 278 (100)	92 409 (100)	5.3	8.5	8.7
Women								
1st quartile (poorest)	3735 (40.1)	82.6	17 788 (39.6)	28 260 (38.9)	32 706 (43.7)	4.8	7.6	8.8
2nd quartile	2195 (23.5)	83.7	11 060 (24.7)	18 099 (24.9)	17 680 (23.6)	5	8.2	8.1
3rd quartile	1877 (20.1)	84.5	9099 (20.3)	15 241 (21.0)	14 265 (19.0)	4.9	8.1	7.6
4th quartile (richest)	1516 (16.3)	86	6921 (15.4)	11 038 (15.2)	10 255 (13.7)	4.6	7.3	6.8
Total	9323 (100)	83.8	44 868 (100)	72 638 (100)	74 906 (100)	4.8	7.8	8

a Number in brackets is the percentage within sex-group.

The estimates based on the standard life table approach result in different patterns. Not only are the average YLL considerably higher (8.7 for men and 8 for women), but there is now a visible income gradient in YLL. This gradient is solely driven by the lower average age at death (as life expectancy by age is equal across income groups in this approach by construction).

The estimates based on income-stratified life tables lead to a similar average for YLL as for the standard life tables. However, as life expectancy increases with income, the average YLL for the lowest income group decreases from 9.4/8.8 years, based on the standard approach, to 8.5/7.6 years, based on the stratified approach, respectively for men/women. For the highest income group, the average YLL increase from 7.9/6.8 to 8.6/7.3 years. The estimates of the income-stratified life tables are considerably higher than in our approach controlling for pre-existing health.

Based on the prediction model, the total number of YLL due to COVID-19 deaths in The Netherlands in 2020 is 100 743, compared with 162 916 based on income-stratified life tables and 167 315 based on standard life tables. There is a strong income gradient in total YLL: the poorest income quartile bears 37% of the total YLL for men and 40% for women. For the richest income quartile this number is between 15% and 16%.

## Discussion

Using individual-level administrative data for the Dutch 50+ population, we take pre-existing health problems into account to calculate YLL to COVID-19, and find 100 743 YLL in 2020, corresponding to 5.3 YLL for men and 4.8 for women on average per COVID-19 death. Total YLL are strongly concentrated among the lower incomes, with the poorest income quartile accounting for 40% of all YLL. On the individual level, there are little differences in YLL. Despite the considerably lower average age at death among the lower incomes, average YLL are similar across incomes. This suggests that, conditional on age, individuals with low incomes dying from COVID-19 were on average in poorer health than those with higher incomes.

### Strengths of the study

Our results provide several new insights. First, we confirm the relevance of correcting for pre-existing health when investigating the burden of disease from COVID-19 mortality. Wouterse *et al.*^17^ estimated the YLL due to COVID-19 in The Netherlands in 2020 as well. They arrived at similar estimates of the average YLL for men and women as our study. It is noteworthy that estimates using aggregate-level data, such as those in Wouterse *et al*.,^17^ arrived at comparable estimates of YLL without the use of individual-level data. This suggests that, although individual-based estimates rely on considerably less assumptions, accounting for prior health when estimating YLL is possible in settings without such data. However, they had to rely on existing aggregate-level estimates of disease-specific excess mortalities, whereas we were able to use detailed administrative data for the entire Dutch population. Three other studies used individual-level data for an entire population: in Sweden,^14^ in Hungary^15^ and in Scotland and Wales.^16^ Ebeling *et al*.^14^ used Swedish administrative data to calculate care-specific life expectancy where types of care fall into no care, home care or care home. They found that unadjusted (standard) YLL are 8.6 for men and 7.5 for women, whereas adjusted YLL are, respectively, 6.6 and 5.4 per COVID-19 death over the year 2020. However, care data were only available for ages 70+. Ferenci^15^ found unadjusted average YLL to be 10.5 per COVID-19 death in Hungary as of May 2021. After correcting for 11 comorbidities, the average number of YLL decreased to 9.2. Hanlon *et al.*^16^ used likely combinations of long-term conditions (LTC) among those who die of COVID-19 in Italy and then used UK health care data to estimate life expectancy for each age, sex and LTC combination group. They found unadjusted YLL to be 14 for men and 12 for women, whereas they fell to 11.6 and 9.4 when adjusted for health. These studies make use of individual-level data, but they do not account for all possible comorbidities, nursing home care status and income simultaneously, which might explain their higher estimates of YLL.

Second, the results show that correcting for pre-existing health status is also important when analysing income differences in the burden of disease from COVID-19 mortality. Available studies^4–6^ rely on population-wide estimates of remaining life expectancy, ignoring potential income differences in health among those who died of COVID-19. We found that although poorer individuals died on average at an earlier age, they lost a similar amount of life-years per COVID-19 death as richer individuals. An alternative estimation—based on standard life expectancy and therefore ignoring age-specific differences in health across income groups—resulted in a strong income gradient in average YLL per COVID-19 death, driven by the younger age at death of poor individuals.

Third, we found that, even when accounting for pre-existing differences in health on the individual level, the aggregate burden of disease from COVID-19 mortality was strongly concentrated among the poorest income quartiles. Given that average YLL per COVID-19 death are similar across income groups, this suggests that the unequal distribution of YLL is mainly driven by the larger number of COVID-19 deaths among the lower-income group.

### Limitations

There are several limitations to our study. First, we do (naturally) not know the counterfactual remaining life expectancy of those dying of COVID-19, and thus have had to rely on estimates. These estimates are based on associations between age, sex, income, health and mortality in 2019, and thus depend on a continuity in the relationship between mortality risk and the chosen covariates over time (from 2019 to 2020), and also necessarily ignore any changes in these relationships which might have occurred in 2020 if COVID-19 had not happened. Moreover, our life table approach relies on the assumption that an individual who dies of COVID-19 at a certain age would have been faced with the same average mortality probability at another age as the individuals of that other age in the same sex-income group who also died of COVID-19. This assumption, which is necessary to calculate life table-based estimates of YLL, ignores potentially important heterogeneity among the individuals who died of COVID-19, for instance between individuals with long-lasting chronic conditions and individuals who were vulnerable to COVID-19 mortality because of a temporarily weakened state caused by short-term health problems. An alternative estimation method would be to estimate survival models using data spanning multiple years. However, a drawback of that approach would be that estimates of YLL would be either highly dependent on extrapolation or old data on baseline health.^26^

Second, to be able to use data on the entire Dutch population, we had to rely on outpatient medication use and nursing home admissions as a proxy for health status. Although this is already an improvement over previous studies that had to rely on limited indicators of health,^14–16^ these indicators might not fully capture all relevant dimensions of health and do not account for the number of prescriptions of the same drug. Moreover, having a drug prescribed does not ensure compliance to the prescription, which could cause bias and overestimation in mortality probabilities. This could have potentially further lowered YLL calculated by the model, although previous evidence shows high drug compliance in The Netherlands.^27^ In addition, indicators of health care use might reflect income differences in access to care rather than in health. Given that income differences in health care access in The Netherlands are small,^28^ we think that the risk of underestimation of the prior health conditions among the poorest group, which would result from lower access for these groups, is limited.

Third, we focused on the health burden of COVID-19 mortality in 2020 only. With the availability of COVID-19 vaccines and other changes in the societal response to the pandemic, the income distribution of the health burden of COVID-19 mortality might be different in later years. As more data becomes available, extending the approach to more recent years therefore is an important step for future research. Similarly, future studies should include the (long-term) health burden of non-lethal COVID-19 infections. Additionally, whereas our investigation focused on one dimension of socioeconomic inequality (income inequality), it would be valuable to also explore other dimensions such as education, occupation and wealth, either individually or jointly. However, obtaining administrative records on these additional dimensions for an entire population might be challenging.

Finally, similar to other YLL studies, we made use of period life tables that do not represent the mortality probabilities that individuals will be subjected to throughout their lives, but rather the mortality probabilities observed in a population at various ages in a single year. They do not account for trends in mortality caused by, for instance, health care advances (past and future) and birth cohort differences in lifestyle. To what extent that assumption biases our findings is difficult to determine. Compared with other studies on YLL due to COVID-19, we believe our study to be less affected, given that we condition on health and thus capture part of these cohort differences.

### Policy implications

Our findings highlight the importance of the interaction between structural income differences in health and exposure to specific health threats, such as the COVID-19 pandemic. It is sometimes argued that by correcting for pre-existing differences in health to estimate the burden of disease of COVID-19, we might lose focus from the structural factors that shape these differences in the first place (e.g. Devleesschauwer *et al*.^29^). Rather than not correcting for pre-existing health, we would argue that the interplay between existing inequalities and unequal exposure to COVID-19 should be made explicit, to allow policy makers to take distributional concerns into account when shaping health policy. Such an inherently normative assessment benefits from having an accurate estimation of the counterfactual in terms of YLL as a starting point.

We found that the unequal distribution of the aggregate YLL due to COVID-19 by income is driven by the larger number of deaths in the lower-income groups. Our results indicate that possibly a larger share of lower-income individuals was vulnerable to the severe health outcomes of a COVID-19 infection due to poorer initial health, probably partly due to an unhealthier lifestyle.^30^^,^^31^ In addition, individuals with a lower income were more exposed to infections by the virus, for instance due to different working and living conditions.^32^ As a matter of fact, low-income individuals are often aggregated in major Dutch cities where the population density is higher, and therefore incur a higher risk of COVID-19.^33^

## Conclusion

Taking prior health into account leads to considerably lower estimates of both the total disease burden and the average YLL per COVID-19 death, compared with an approach based on the income-stratified or standard population life tables. Even though the average YLL per COVID-19 death are similar between poorer and richer income quartiles, our estimates re-emphasize the unequal distribution of the burden of disease from COVID-19 mortality. Poorer individuals died of COVID-19 at a younger age on average, and in the aggregate, the total YLL is strongly skewed towards the poorest income quartile. Curated policies better adapted to the socioeconomic circumstances of low-income groups should be considered when shaping the policy response to future public health threats.

## Ethics approval

The project has received approval for data access and results dissemination from the Centraal Bureau voor de Statistiek (Statistics Netherlands). No further ethical approval is required.

## Supplementary Material

dyad159_Supplementary_Data

## Data Availability

The data underlying this article cannot be shared publicly due to privacy reasons. The data can be made available for researchers by the Centraal Bureau voor de Statistiek (Statistics Netherlands) through a remote access environment. To access the data, researchers must submit a research proposal to Statistics Netherlands [microdata@cbs.nl] and pay a fee. The authors are willing to assist, contact Jawa Issa [issa@eshpm.eur.nl].
