# Supplementary material for: Quantifying income inequality in years of life lost to COVID-19: a prediction model approach using Dutch administrative data
Source: Int J Epidemiol. 2023 Dec 11;53(1):dyad159. doi: 10.1093/ije/dyad159 (PMC10859130; doi:10.1093/ije/dyad159)
Supplement: dyad159_Supplementary_Data [file dyad159_supplementary_data.docx]

**Quantifying income inequality in years of life lost to COVID-19: a prediction model approach using Dutch administrative data**

**Supplementary materials**

[Supplementary data: Sample selection 2](#_Toc148100444)

[Supplementary methods: Life table calculations 3](#_Toc148100445)

[Table S1. Logistic regression coefficients (inputs for the prediction of probability of death in 2020) for various subgroups based on the residents of the Netherlands on January 1st, 2019 4](#_Toc148100446)

[Table S2. Percentage use of various medications (ATC-4 first level codes) and nursing home attendance within sex by income quartile (Q1, poorest and Q4, richest) in the Netherlands in 2019 12](#_Toc148100447)

[Figure S2. Average income by age, sex, and income quartile for residents of the Netherlands aged 50+ on January 1^st^, 2020 13](#_Toc148100448)

[Table S3. COVID-19 deaths by age, sex, income quartile, excluding nursing home residents, in the Netherlands in 2020 13](#_Toc148100449)

[Table S4. COVID-19 deaths by age, sex, income quartile for nursing home residents in the Netherlands in 2020 14](#_Toc148100450)

[Figure S3. Average probability of death by age for men (A) and women (B) who die of COVID-19 as calculated using predictions of the regression model, and for men (C) and women (D) as calculated using income-stratified life tables for individuals who die of COVID-19 in the Netherlands in 2020. Numbers are smoothed using cubic splines in accordance with anonymity requirements of Statistics Netherlands. 15](#_Toc148100451)

[Figure S4. Remaining life expectancy by age for men (A) and women (B) who die of COVID-19 as calculated using predictions of the regression model, and for men (C) and women (D) as calculated using income-stratified life tables for individuals who die of COVID-19 in the Netherlands in 2020. Numbers are smoothed using cubic splines in accordance with anonymity requirements of Statistics Netherlands. 16](#_Toc148100452)

[Figure S5. YLL by age for men (A) and women (B) who die of COVID-19 as calculated using predictions of the regression model, for men (C) and women (D) as calculated using income-stratified life tables, and for men (E) and women (F) as calculated using standard life tables for individuals who die of COVID-19 in the Netherlands in 2020. Numbers are smoothed using cubic splines in accordance with anonymity requirements of Statistics Netherlands. 17](#_Toc148100453)

**Supplementary data: Sample selection**

1. 2019 sample
2. 2020 sample

Figure S1. Sample selection for 2019 (Panel A) and 2020 (Panel B)

Using administrative datasets, we perform a sample selection which is described in Fig I. We train the prediction model on the 2019 sample (Panel A) and use the coefficients to predict mortality probabilities for those who die of COVID-19 in 2020 (sample in Panel B) – regression coefficients are available in Supplementary Table I.

We construct the 2019 sample by first identifying all residents of the Netherlands on January 1^st^ and then restricting the analysis to those who are 50 years or older. Next, we link individuals to their disposable income. We lose around 0.5% of the sample during this step for those with unavailable income data. Finally, we link medication use, nursing home attendance and death registry information for those with available data.

For the 2020 sample, we follow a similar process. However, we ultimately condition on cause of death and keep only individuals who die of COVID-19 throughout the year.

**Supplementary methods: Life table calculations**

We estimate YLL in three steps. First, we calculate the mortality probability for those who die of COVID-19 using the prediction model trained on the 2019 sample. We obtain estimates for the counterfactual probability of death for each COVID-19 decedent in the 2020 sample. Then, we average out their risk across covariates. Equation (1) gives the average probability of all-cause death conditional on age (*x*), sex (*s*), and income (*i*), with *d^COVID-19^* being the COVID-19 deaths, *q^j^* being the predicted probability of death, and *n* being the number of individuals in the age, sex, income group. We smooth over the resulting age patterns with cubic splines.

$q_{x,s,i}= \frac{\sum_{j\in R(d_{x,s,i}^{COVID-19})} q_{x,s,i}^{j}}{n_{x,s,i}}$ (1)

Next, we built life tables for each sex and income quartile using the equations (2) to (7), including only individuals who die of COVID-19. We first convert the probabilities into mortality rates (2) and then calculate the number of survivors at age *x* for an initial cohort of 100 000 (*l_x,s,i_* with *l_50,s,i_*=100 000) individuals (3).

$m_{x,s,i}=-ln(1-q_{x,s,i})$ (2)

$l_{x,s,i}=l_{50}\cdot\prod_{j=50}^{j<x} (1-q_{j,s,i})$ (3)

Using (4), we find the age-at-death distribution for the cohort.

$d_{x,s,i}=l_{x,s,i}\cdot q_{x,s,i}$ For the 100+ category, *d_100+_=l_100+_*. (4)

Then, the person-years lived in the age interval are: $L_{x,s,i}=l_{x,s,i}+0.5d_{x,s,i}$ (5)

For the 100+ category, *L_x,s,i_=l_x,s,i_/m_x,s,i_*. With this information, we find *T_x,s,i_*, the total number of person-years lived above age *x* (6). With *L_x,s,i_* and *T_x,s,i_*, we can calculate the remaining life expectancy at every age (7).

$T_{x,s,i}= \sum_{j=x}^{99} L_{j,s,i}+ L_{100+,s,i}$ (6)

$e_{x,s,i}=\frac{T_{x,s,i}}{l_{x,s,i}}$ With $e_{100+,s,i}=\frac{1}{m_{100+,s,i}}$ (7)

As a last step, we use remaining life expectancy by sex and income quartile to calculate YLL to COVID-19 and average YLL per COVID-19 death via expressions (8) and (9) respectively.

${YLL}_{x,s,i}=e_{x,s,i}\cdot d_{x,s,i}^{COVID-19}$ (8)

$\bar{{YLL}_{s,i}}=\frac{\sum_{x=50}^{100+} e_{x,s,i}\cdot d_{x,s,i}^{COVID-19}}{d_{s,i}^{COVID-19}}$ (9)

**Table S1. Logistic regression coefficients (inputs for the prediction of probability of death in 2020) for various subgroups based on the residents of the Netherlands on January 1st, 2019**

|  | Men 50+ non-Nursing home | Women 50+ non-Nursing home | Men 50+ Nursing home | | Women 50+ Nursing home |
| --- | --- | --- | --- | --- | --- |
|  | (1) | (2) | (3) | | (4) |
| Log income | 0.869 *** (0.005) | 0.889 *** (0.007) | 1.3 *** (0.036) | | 1.067 *** (0.019) |
| Age |  |  |  | |  |
| 51 | 1.152 * (0.094) | 1.261 ** (0.118) | 0.983 (0.303) | | 1.637  (0.64) |
| 52 | 1.117 (0.091) | 1.232 ** (0.115) | 1.256 (0.368) | | 2.471 ** (0.889) |
| 53 | 1.152 * (0.093) | 1.319 *** (0.121) | 1.213 (0.353) | | 1.615 (0.612) |
| 54 | 1.423 *** (0.109) | 1.411 *** (0.127) | 1.115 (0.324) | | 2.037 * (0.745) |
| 55 | 1.534 *** (0.116) | 1.644 *** (0.143) | 1.235 (0.354) | | 2.151 ** (0.774) |
| 56 | 1.628 *** (0.122) | 1.789 *** (0.154) | 1.257  (0.36) | | 1.971 * (0.725) |
| 57 | 1.712 *** (0.127) | 1.828 *** (0.156) | 2.27 *** (0.585) | | 2.398 ** (0.851) |
| 58 | 2.021 *** (0.146) | 2.115 *** (0.177) | 2.316 *** (0.595) | | 3.226 *** (1.121) |
| 59 | 2.19 *** (0.156) | 2.106 *** (0.176) | 1.497 (0.409) | | 4.831 *** (1.612) |
| 60 | 2.102 *** (0.151) | 2.393 *** (0.197) | 2.808 *** (0.709) | | 4.58 *** (1.537) |
| 61 | 2.528 *** (0.177) | 2.589 *** (0.211) | 2.665 *** (0.673) | | 4.493 *** (1.508) |
| 62 | 2.443 *** (0.172) | 2.799 *** (0.226) | 2.583 *** (0.656) | | 3.983 *** (1.346) |
| 63 | 2.829 *** (0.195) | 3.135 *** (0.25) | 3.086 *** (0.766) | | 4.035 *** (1.369) |
| 64 | 2.96 *** (0.203) | 3.154 *** (0.251) | 3.518 *** (0.861) | | 4.092 *** (1.377) |
| 65 | 3.327 *** (0.225) | 3.287 *** (0.261) | 4.446 *** (1.063) | | 4.443 *** (1.482) |
| 66 | 3.399 *** (0.23) | 3.64 *** (0.286) | 4.325 *** (1.034) | | 6.323 *** (2.051) |
| 67 | 3.661 *** (0.246) | 4.24 *** (0.329) | 4.766 *** (1.132) | | 6.959 *** (2.256) |
| 68 | 4.237 *** (0.281) | 3.969 *** (0.309) | 5.125 *** (1.212) | | 7.666 *** (2.462) |
| 69 | 4.496 *** (0.296) | 4.812 *** (0.368) | 4.829 *** (1.141) | | 8.357 *** (2.659) |
| 70 | 4.599 *** (0.302) | 4.8 *** (0.366) | 6.457 *** (1.498) | | 9.774 *** (3.096) |
| 71 | 5.034 *** (0.327) | 5.195 *** (0.392) | 8.206 *** (1.875) | | 9.081 *** (2.863) |
| 72 | 5.453 *** (0.352) | 5.615 *** (0.421) | 9.707 *** (2.197) | | 10.784 *** (3.379) |
| 73 | 5.933 *** (0.389) | 6.041 *** (0.461) | 9.014 *** (2.073) | | 11.888 *** (3.737) |
| 74 | 6.43 *** (0.419) | 6.218 *** (0.472) | 10.922 *** (2.476) | | 12.851 *** (4.012) |
| 75 | 6.756 *** (0.44) | 7.096 *** (0.535) | 11.486 *** (2.611) | | 12.476 *** (3.889) |
| 76 | 7.281 *** (0.475) | 7.037 *** (0.534) | 12.611 *** (2.85) | | 15.797 *** (4.902) |
| 77 | 7.75 *** (0.506) | 7.972 *** (0.603) | 12.994 *** (2.94) | | 13.545 *** (4.2) |
| 78 | 8.845 *** (0.573) | 7.988 *** (0.603) | 13.791 *** (3.092) | | 17.43 *** (5.371) |
| 79 | 10.084 *** (0.653) | 9.367 *** (0.702) | 15.787 *** (3.528) | | 14.93 *** (4.601) |
| 80 | 10.376 *** (0.673) | 10.214 *** (0.765) | 17.092 *** (3.804) | | 16.961 *** (5.218) |
| 81 | 11.688 *** (0.759) | 11.223 *** (0.84) | 17.707 *** (3.947) | | 18.001 *** (5.53) |
| 82 | 12.477 *** (0.811) | 12.304 *** (0.919) | 16.87 *** (3.75) | | 17.398 *** (5.337) |
| 83 | 13.967 *** (0.909) | 13.338 *** (0.995) | 19.081 *** (4.229) | | 19.161 *** (5.872) |
| 84 | 16.281 *** (1.06) | 14.749 *** (1.1) | 19.417 *** (4.308) | | 19.274 *** (5.902) |
| 85 | 17.62 *** (1.154) | 16.579 *** (1.236) | 22.728 *** (5.031) | | 20.494 *** (6.27) |
| 86 | 19.492 *** (1.28) | 19.429 *** (1.445) | 20.618 *** (4.565) | | 20.09 *** (6.143) |
| 87 | 23.217 *** (1.531) | 21.311 *** (1.589) | 22.9 *** (5.066) | | 20.867 *** (6.378) |
| 88 | 24.651 *** (1.644) | 23.733 *** (1.772) | 21.748 *** (4.82) | | 22.376 *** (6.837) |
| 89 | 28.197 *** (1.91) | 27.99 *** (2.1) | 24.828 *** (5.52) | | 24.737 *** (7.558) |
| 90 | 31.465 *** (2.173) | 30.959 *** (2.338) | 27.327 *** (6.083) | | 26.338 *** (8.047) |
| 91 | 33.299 *** (2.365) | 34.444 *** (2.631) | 26.128 *** (5.838) | | 27.703 *** (8.468) |
| 92 | 40.621 *** (2.956) | 42.642 *** (3.28) | 28.53 *** (6.408) | | 28.019 *** (8.568) |
| 93 | 39.988 *** (3.084) | 46.447 *** (3.644) | 26.231 *** (5.949) | | 30.053 *** (9.197) |
| 94 | 48.77 *** (3.986) | 50.084 *** (4.016) | 34.098 *** (7.831) | | 34.149 *** (10.456) |
| 95 | 55.902 *** (4.874) | 60.24 *** (4.973) | 32.511 *** (7.545) | | 34.258 *** (10.509) |
| 96 | 49.971 *** (5.182) | 73.434 *** (6.327) | 31.298 *** (7.433) | | 35.949 *** (11.051) |
| 97 | 59.265 *** (6.921) | 81.621 *** (7.573) | 43.73 *** (10.981) | | 40.119 *** (12.375) |
| 98 | 85.95 *** (11.371) | 77.736 *** (8.04) | 36.342 *** (9.517) | | 41.359 *** (12.825) |
| 99 | 105.148 *** (17.937) | 83.226 *** (9.934) | 34.602 *** (9.73) | | 44.022 *** (13.816) |
| 100+ | 107.052 *** (18.292) | 115.927 *** (12.53) | 34.315 *** (9.409) | | 51.534 *** (15.994) |
| Outpatient medication | | | |  |  |
| Alimentary tract and metabolism | | | |  |  |
| A01 | 0.974 (0.049) | 0.984 (0.059) |  | |  |
| A02 | 1.066 *** (0.012) | 0.991 (0.012) |  | |  |
| A03 | 1.908 *** (0.035) | 1.803 *** (0.028) |  | |  |
| A04 | 4.255 *** (0.115) | 4.69 *** (0.118) |  | |  |
| A05 | 1.229 ** (0.105) | 1.571 *** (0.11) |  | |  |
| A06 | 1.335 *** (0.015) | 1.324 *** (0.015) |  | |  |
| A07 | 1.288 *** (0.03) | 1.234 *** (0.028) |  | |  |
| A09 | 1.718 *** (0.084) | 2.366 *** (0.131) |  | |  |
| A10 | 1.353 *** (0.016) | 1.399 *** (0.019) |  | |  |
| A11 | 1.216 *** (0.014) | 1.109 *** (0.013) |  | |  |
| A12 | 1.169 *** (0.019) | 1.045 *** (0.014) |  | |  |
| A14 | 0.211 (0.22) | 0.767 (0.389) |  | |  |
| A16 | 0.954 (0.341) | 1.134 (0.288) |  | |  |
| Blood and blood forming organs | | | |  |  |
| B01 | 1.344 *** (0.016) | 1.444 *** (0.018) |  | |  |
| B02 | 1.839 *** (0.064) | 1.863 *** (0.07) |  | |  |
| B03 | 1.338 *** (0.019) | 1.334 *** (0.019) |  | |  |
| B05 | 1.537 *** (0.062) | 1.939 *** (0.076) |  | |  |
| B06 | 0.721 (0.757) |  |  | |  |
| Cardiovascular system | | | |  |  |
| C01 | 1.108 *** (0.015) | 1.039 *** (0.015) |  | |  |
| C02 | 1.093 ** (0.039) | 1.106 ** (0.045) |  | |  |
| C03 | 1.494 *** (0.016) | 1.348 *** (0.015) |  | |  |
| C04 | 1.409 ** (0.236) | 1.011 (0.203) |  | |  |
| C05 | 0.813 *** (0.06) | 0.858 * (0.07) |  | |  |
| C07 | 1.127 *** (0.012) | 1.098 *** (0.012) |  | |  |
| C08 | 0.946 *** (0.011) | 0.981 (0.012) |  | |  |
| C09 | 0.933 *** (0.01) | 0.974 ** (0.011) |  | |  |
| C10 | 0.765 *** (0.008) | 0.788 *** (0.009) |  | |  |
| Dermatologicals | | | |  |  |
| D01 | 0.925 *** (0.015) | 1.004 (0.017) |  | |  |
| D02 | 1.247 *** (0.018) | 1.245 *** (0.017) |  | |  |
| D03 | 1.329 ** (0.183) | 2.014 *** (0.247) |  | |  |
| D04 |  | 20.857 ** (26.654) |  | |  |
| D05 | 0.96  (0.044) | 1.161 *** (0.06) |  | |  |
| D06 | 1.081 *** (0.019) | 1.075 *** (0.019) |  | |  |
| D07 | 0.867 *** (0.011) | 0.81 *** (0.011) |  | |  |
| D08 | 1.386 ** (0.195) | 1.11  (0.181) |  | |  |
| D09 | 1.287 (0.213) | 1.355 ** (0.19) |  | |  |
| D10 | 0.839 ** (0.064) | 1.003 (0.086) |  | |  |
| D11 | 0.768 *** (0.037) | 0.793 *** (0.039) |  | |  |
| Genito-urinary system and sex hormones | | | |  |  |
| G01 | 0.714 (0.269) | 0.925 ** (0.037) |  | |  |
| G02 | 0.735 (0.165) | 1.586 * (0.436) |  | |  |
| G03 | 0.925 (0.048) | 0.687 *** (0.02) |  | |  |
| G04 | 0.898 *** (0.01) | 1.037 (0.026) |  | |  |
| Systemic hormonal preparations, excluding sex hormones and insulins | | | |  |  |
| H01 | 1.456 *** (0.104) | 1.476 *** (0.126) |  | |  |
| H02 | 1.588 *** (0.019) | 1.625 *** (0.02) |  | |  |
| H03 | 1.067 *** (0.025) | 0.931 *** (0.014) |  | |  |
| H04 | 1.22 *** (0.089) | 1.427 *** (0.114) |  | |  |
| H05 | 1.19 ** (0.105) | 1.295 *** (0.09) |  | |  |
| Antiinfectives for systemic use | | | |  |  |
| J01 | 1.341 *** (0.014) | 1.303 *** (0.014) |  | |  |
| J02 | 1.244 *** (0.051) | 1.251 *** (0.048) |  | |  |
| J04 | 0.637 *** (0.072) | 0.467 *** (0.072) |  | |  |
| J05 | 0.923 ** (0.034) | 1.086 ** (0.043) |  | |  |
| J06 | 0.747 (0.169) | 0.895 (0.211) |  | |  |
| J07 | 0.882 *** (0.032) | 0.878 *** (0.038) |  | |  |
| Antineoplastic and immunomodulating agents | | | |  |  |
| L01 | 0.915 ** (0.033) | 0.779 *** (0.038) |  | |  |
| L02 | 2.459 *** (0.057) | 2.394 *** (0.059) |  | |  |
| L03 | 0.993 (0.068) | 0.876 ** (0.056) |  | |  |
| L04 | 0.899 *** (0.029) | 0.887 *** (0.029) |  | |  |
| Musculo-skeletal system | | | |  |  |
| M01 | 0.841 *** (0.011) | 0.823 *** (0.011) |  | |  |
| M02 |  | 0.412 (0.439) |  | |  |
| M03 | 1.54 *** (0.088) | 1.588 *** (0.117) |  | |  |
| M04 | 1.136 *** (0.019) | 1.205 *** (0.03) |  | |  |
| M05 | 1.114 *** (0.026) | 1.054 *** (0.018) |  | |  |
| Nervous system | | | |  |  |
| N01 | 0.83 *** (0.025) | 0.736 *** (0.022) |  | |  |
| N02 | 1.356 *** (0.016) | 1.274 *** (0.015) |  | |  |
| N03 | 1.24 *** (0.022) | 1.21 *** (0.022) |  | |  |
| N04 | 1.316 *** (0.036) | 1.078 ** (0.033) |  | |  |
| N05 | 1.923 *** (0.029) | 1.721 *** (0.024) |  | |  |
| N06 | 1.171 *** (0.017) | 1.107 *** (0.015) |  | |  |
| N07 | 1.123 *** (0.033) | 0.986 (0.026) |  | |  |
| Antiparasitic products, insecticides and repellents | | | |  |  |
| P01 | 0.844 *** (0.039) | 0.9 *** (0.034) |  | |  |
| P02 | 0.992 (0.485) | 1.016 (0.466) |  | |  |
| P03 | 0.806 (0.151) | 0.885 (0.196) |  | |  |
| Respiratory system | | | |  |  |
| R01 | 0.714 *** (0.012) | 0.671 *** (0.012) |  | |  |
| R03 | 1.287 *** (0.015) | 1.277 *** (0.016) |  | |  |
| R05 | 0.965 ** (0.017) | 0.87 *** (0.015) |  | |  |
| R06 | 0.872 *** (0.017) | 0.88 *** (0.016) |  | |  |
| Sensory organs | | | |  |  |
| S01 | 0.89 *** (0.01) | 0.829 *** (0.009) |  | |  |
| S02 | 0.804 *** (0.019) | 0.862 *** (0.023) |  | |  |
| Various |  |  |  | |  |
| V01 | 0.26 * (0.185) | 1.246 (0.476) |  | |  |
| V03 | 2.297 *** (0.09) | 2.795 *** (0.145) |  | |  |
| V04 | 1.829 (0.809) | 1.322 (0.713) |  | |  |
| V07 | 1.091 (0.125) | 0.949 (0.14) |  | |  |
| Not disclosed | | | |  |  |
| Y | 3.58 *** (0.056) | 3.35 *** (0.049) |  | |  |
| Constant | 0.007 *** (0.001) | 0.004 *** (0) | 0.002 *** (0.001) | | 0.009 *** (0.003) |
| N | 3257404 | 3466517 | 57517 | | 104413 |
| Pseudo r-squared | 0.241 | 0.238 | 0.127 | | 0.055 |

*** p<.01, ** p<.05, * p<.1, standard errors between parentheses

**Table S2. Percentage use of various medications (ATC-4 first level codes) and nursing home attendance within sex by income quartile (Q1, poorest and Q4, richest) in the Netherlands in 2019**

|  | Men | | | | Women | | | |
| --- | --- | --- | --- | --- | --- | --- | --- | --- |
|  | Q1 | Q2 | Q3 | Q4 | Q1 | Q2 | Q3 | Q4 |
| A Alimentary tract and metabolism | 19.34 | 16.10 | 14.07 | 12.24 | 23.71 | 20.72 | 18.16 | 15.53 |
| B Blood and blood forming organs | 8.72 | 8.29 | 7.59 | 6.90 | 7.25 | 6.87 | 6.16 | 5.37 |
| C Cardiovascular system | 31.00 | 29.84 | 27.33 | 24.27 | 27.94 | 27.11 | 24.15 | 20.42 |
| D Dermatologicals | 7.92 | 7.35 | 7.16 | 7.17 | 9.18 | 8.78 | 8.39 | 8.11 |
| G Genito-urinary system and sex hormones | 2.64 | 2.56 | 2.55 | 2.53 | 1.77 | 1.71 | 1.69 | 1.78 |
| H Systemic hormonal preparations, excluding sex hormones and insulins | 3.09 | 2.87 | 2.53 | 2.19 | 5.25 | 5.06 | 4.55 | 3.95 |
| J Antiinfectives for systemic use | 5.98 | 5.76 | 5.52 | 5.54 | 7.65 | 7.63 | 7.38 | 7.24 |
| L Antineoplastic and immunomodulating agents | 0.72 | 0.81 | 0.82 | 0.84 | 0.92 | 1.03 | 1.03 | 1.02 |
| M Musculo-skeletal system | 5.58 | 5.47 | 5.05 | 4.58 | 6.31 | 6.02 | 5.70 | 5.24 |
| N Nervous system | 9.34 | 6.40 | 5.27 | 4.34 | 12.57 | 10.25 | 8.66 | 7.19 |
| P Antiparasitic products, insecticides and repellents | 0.20 | 0.18 | 0.18 | 0.18 | 0.42 | 0.37 | 0.36 | 0.35 |
| R Respiratory system | 8.10 | 7.53 | 7.17 | 6.92 | 10.99 | 10.13 | 9.42 | 8.80 |
| S Sensory organs | 4.20 | 4.02 | 3.98 | 4.01 | 5.55 | 5.32 | 5.26 | 5.31 |
| V Various | 0.10 | 0.07 | 0.07 | 0.06 | 0.07 | 0.05 | 0.05 | 0.04 |
| Y Not disclosed | 0.48 | 0.32 | 0.26 | 0.22 | 0.56 | 0.43 | 0.36 | 0.30 |
| Nursing home | 1.08 | 0.27 | 0.22 | 0.16 | 1.58 | 0.51 | 0.43 | 0.36 |

**Figure S2. Average income by age, sex, and income quartile for residents of the Netherlands aged 50+ on January 1^st^, 2020**

**Table S3. COVID-19 deaths by age, sex, income quartile, excluding nursing home residents, in the Netherlands in 2020**

|  |  | **50 to 59** | **60 to 69** | **70 to 79** | **80 to 89** | **90+** | **Total** |
| --- | --- | --- | --- | --- | --- | --- | --- |
|  | Q1 (poorest) | 103 | 307 | 847 | 894 | 179 | 2330 |
| **Men** | Q2 | 54 | 209 | 663 | 851 | 216 | 1993 |
|  | Q3 | 46 | 168 | 508 | 640 | 177 | 1539 |
|  | Q4 (richest) | 26 | 112 | 390 | 555 | 154 | 1237 |
|  | Q1 (poorest) | 61 | 178 | 399 | 550 | 162 | 1350 |
| **Women** | Q2 | 32 | 99 | 334 | 536 | 209 | 1210 |
|  | Q3 | 22 | 74 | 229 | 462 | 215 | 1002 |
|  | Q4 (richest) | 16 | 40 | 140 | 364 | 226 | 786 |

**Table S4. COVID-19 deaths by age, sex, income quartile for nursing home residents in the Netherlands in 2020**

|  |  | **70 to 79** | **80 to 89** | **90+** | **Total** |
| --- | --- | --- | --- | --- | --- |
|  | Q1 (poorest) | 439 | 732 | 284 | 1455 |
| **Men** | Q2 | 182 | 407 | 147 | 736 |
|  | Q3 | 130 | 354 | 141 | 625 |
|  | Q4 (richest) | 94 | 285 | 122 | 501 |
|  | Q1 (poorest) | 406 | 1118 | 731 | 2255 |
| **Women** | Q2 | 123 | 453 | 392 | 968 |
|  | Q3 | 108 | 411 | 345 | 864 |
|  | Q4 (richest) | 68 | 326 | 328 | 722 |

# **Figure S3. Average probability of death by age for men (A) and women (B) who die of COVID-19 as calculated using predictions of the regression model, and for men (C) and women (D) as calculated using income-stratified life tables for individuals who die of COVID-19 in the Netherlands in 2020. Numbers are smoothed using cubic splines in accordance with anonymity requirements of Statistics Netherlands.**

**Figure S4. Remaining life expectancy by age for men (A) and women (B) who die of COVID-19 as calculated using predictions of the regression model, and for men (C) and women (D) as calculated using income-stratified life tables for individuals who die of COVID-19 in the Netherlands in 2020. Numbers are smoothed using cubic splines in accordance with anonymity requirements of Statistics Netherlands.**

**Figure S5. YLL by age for men (A) and women (B) who die of COVID-19 as calculated using predictions of the regression model, for men (C) and women (D) as calculated using income-stratified life tables****, and for men (E) and women (F) as calculated using standard life tables for individuals who die of COVID-19 in the Netherlands in 2020.** **Numbers are smoothed using cubic splines in accordance with anonymity requirements of Statistics Netherlands.**
